# Cigarette Consumption Patterns of Non-Smokers, Occasional Smokers, and Daily Smokers in Selected ASEAN Countries

**DOI:** 10.31557/APJCP.2021.22.7.1997

**Published:** 2021-07

**Authors:** Steven T Yen, Andrew K G Tan

**Affiliations:** 1 *International College, Jiangxi University of Finance and Economics, Nanchang City, Jiangxi Province, China. *; 2 *School of Social Sciences, Universiti Sains Malaysia, Minden, Penang, Malaysia. *

**Keywords:** ASEAN, daily smokers, non-smokers, occasional smokers, smoking status

## Abstract

**Background:**

This study investigates the socio-demographic factors associated with smoking status in five Southeast Asian countries: Malaysia, Thailand, Indonesia, Vietnam, and Philippines.

**Methods:**

This cross-sectional study utilizes data of adults ≥15 years who completed the Global Adult Tobacco Surveys. Ordered probit analysis is used to account for the smoking statuses of non-smokers, occasional smokers, and daily smokers.

**Results:**

Malaysian and Vietnamese households with more family members face lower smoking likelihoods than otherwise. Urbanites in Philippines and rural residents in Thailand and Indonesia are more likely to smoke on occasional and daily basis than others. Males are consistently more likely to smoke occasionally or daily and less likely to be non-smokers than females across all countries. Younger middle-age (retiree) individuals aged 30-35 (≥60) years in Malaysia and Thailand exhibit higher (lower) likelihoods to smoke occasionally or daily than their younger cohorts aged 15-29 years. Individuals aged 30 years and above in Indonesia, Vietnam, and Philippines display higher daily smoking propensities than others. Higher education levels dampens smoking likelihoods and increases non-smoking propensities in all countries. Non-government or self-employed workers in all countries are more likely to smoke occasionally or daily than unemployed persons. Being married is associated with higher non-smoking likelihoods in Thailand although this association is not evident in Malaysia.

**Conclusion:**

These findings suggest that a portfolio of targeted interventions is necessary to meet the needs of specific subpopulations within the various countries.

## Introduction

Cigarette smoking is a major modifiable risk factor for a number of non-communicable diseases (Uddin et al., 2020). Nonetheless, adult smoking in countries across Asia have remained high (World Health Organization, 2016; Ritchie and Roser, 2020). While countries such as Malaysia, Thailand, Philippines, and Vietnam experienced declining trends in adult smoking between 2000–2015, prevalence rates remained high as more than 30% of males aged 15 years and above still smoked some type of tobacco product (World Health Organization, 2016). In fact, Indonesian smokers ranked highest among ASEAN countries (Kristina et al., 2015) as smoking prevalence increased from 56.2% in 2000 to 76.2% in 2015 (World Health Organization, 2016).

The health impacts of cigarette smoking is manifested by the elevated risks of smoking-related diseases. One in every 5 cardiovascular diseases (e.g. heart attack, stroke) or 1 million deaths occur annually in the Asia Pacific region as a result of smoking (World Health Organization, 2018). Within selected ASEAN countries, more than one fifth of male deaths in Malaysia (23.1%), Thailand (22.9%), Indonesia (21.4%), Philippines (22.7%), and Vietnam (26.1%) are attributed to tobacco usage (Drope et al., 2018a). In terms of total economic cost of smoking, Indonesia (US$44.6 billion) ranked highest, followed by Thailand (US$6.5 billion), Philippines (US$5.4 billion), Malaysia (US$3.6 billion), and Vietnam (US$3.7 billion) (Drope et al., 2018b).

Studies have investigated cigarette consumption patterns in the ASEAN region (Hammond et al., 2008; Hong and Peltzer, 2019; Pengpid and Peltzer, 2019). The majority of these studies utilized a prevalence-based approach with binary smoking and non-smoking outcomes. However, studies have shown that within those categorized as current smokers, there exists two subgroups consisting of daily and occasional (or intermittent) smokers, whereby both groups exhibit similar and yet divergent characteristics. Compared to non-smokers, both smoking groups share higher smoking-related health risks of depression (Weinberger et al., 2017), respiratory symptoms (An et al., 2009), cancers (Bjerregaard et al., 2006), and mortality (Inoue-Choi et al., 2019). These overall adverse health effects suggest that no proven safe level of smoking exists within daily and occasional tobacco users. Meanwhile, aside from differences in quantum and regularity of cigarettes smoked and nicotine dependency (Shiffman and Paty, 2006), daily and occasional smokers differ in their smoking motivations. Daily smokers are driven by internal cues or negative affectivity (e.g. boredom, stress sensory satisfaction, anxiety, weight and appetite regulation, depression), while occasional smokers are drawn by external or environmental cues (e.g. peer pressure, smoking imagery) (Haight et al., 2012; Shiffman et al., 2014). Since different motivations may dominate for different groups of smokers, daily and occasional smokers may benefit from different policy interventions. On this basis, we extend the analysis by exploiting the unique feature of the datasets available for the countries in question, and use an ordered (vis-à-vis binary) probit model to examine the socio-demographic factors associated with the multi-outcome smoking status of a non-smoker, occasional smoker, and daily smoker.

## Materials and Methods

*Data*

Data were drawn from the Global Adult Tobacco Survey (GATS) of Malaysia, Thailand, Indonesia, Philippines, and Vietnam (World Health Organization 2011a, b, c, 2015a, b). The GATS is a nationally representative household survey of non-institutionalized adults 15 years of age or older based on a standard core questionnaire, sample design, and data collection and management procedures. Country-specific multi-stage stratified cluster sample designs were used. Data collection was conducted in 2011 for Malaysia, Thailand, and Indonesia and in 2015 for Philippines and Vietnam. After removing observations with missing data (0.1 to 0.7%), final samples are 4,220, 20,586, 8,300, 8,985, and 11,603 for Malaysia, Thailand, Indonesia, Vietnam, and Philippines, respectively.

*Outcome variable*

**Figure 1:**
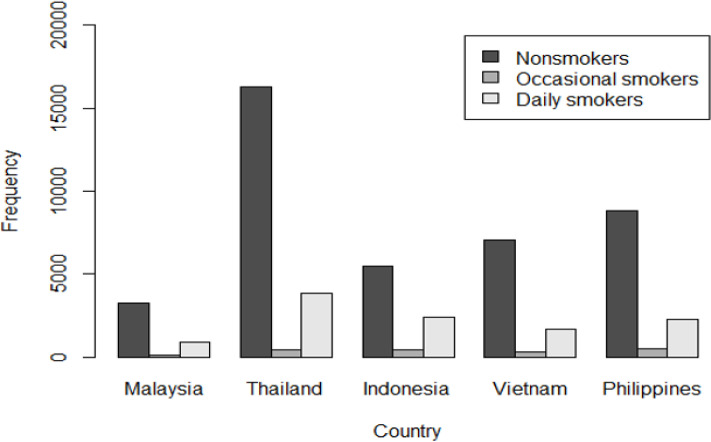
Frequency of Smoking Category by Country

The following question was asked in the survey: “Do you currently smoke tobacco on a daily basis, less than daily, or not at all?” Responses were coded as “daily,” “occasional,” and “non-smokers,” respectively, in our outcome variable. Occasional smokers referred to individuals who used at least one of the smoked tobacco products during the survey period but not on a daily basis regardless of their smoking history. Daily smokers denoted persons who smoked any tobacco product every day during the survey period (World Health Organization 2011b, c, a, 2015a, b; Omar et al., 2013). Frequency of smoking category by country is presented in Figure 1.

*Exposure variables*

From previous literature (Hammond et al., 2008; Tan et al., 2009; Hong and Peltzer, 2019) and based on data availability, exposure variables hypothesized to be associated with smoking status include: Household size, location of residence (Urban), gender (Male), age groups (Age 15−29, Age 30–45, Age 46–59, Age ≥60), education level (Primary School, High School, Tertiary), occupation sector (Unemployed, Government, Non-government), and marital status (data available for Malaysia and Thailand only). Variable definitions are provided in Table 1.

To investigate the roles of socio-demographic factors in determining multiple smoking outcome, we use an ordered probit model. This probability model is characterized by a stochastic process containing a linear index xβ and a random error term u such that (Greene and Hensher, 2010).

Smoking category=not at all                 if -∞<xβ +u≤0

                             =occasional              if 0<xβ+u≤t

                             =daily                       if t<xβ+u≤∞

where x is a vector of explanatory variables, β a conformable vector of parameters, and τ a threshold parameter which, along with the stochastic process xβ+u, delineates categories in the dependent variable. The dependent variable being categorical and not quantitative, parameters (β,τ) are identified only up to a scale and, hence, the error term is assumed to be distributed as standard normal with zero mean and unitary standard deviation. 

Based on (1), category probabilities are F(-xβ) for not non-smokers, F(τ-xβ)-F(-xβ) for occasional smokers, and 1-F(τ-xβ) for daily smokers, where F(∙) is cumulative distribution function of the standard normal. The product of these probabilities, the sample likelihood function, is maximized using the method of maximum likelihood to obtain estimates of the parameters (β,τ) (see Supplementary Appendix Table). Marginal effects, i.e., impacts of changes in a continuous (discrete) variable from smoking outcomes are derived by differentiating (differencing) the aforementioned category probabilities for each sample observation and averaging over the sample. For statistical inference, standard errors of these average marginal effects are calculated by the delta method (Greene and Hensher, 2010).

## Results

*Sample statistics*

Sample statistics of variables are listed in Table 1. The largest proportion of non-smokers are located in Thailand (79%), followed by Vietnam (78%), Malaysia (77%), Philippines (76%), and Indonesia (66%). Indonesia ranked highest in terms of daily (29%) and occasional (5%) smokers.

Average household size for Philippines (4.47) is significantly higher than Thailand (3.07). The urban-rural composition is identical for Malaysia, Indonesia, and Vietnam, although urbanites (61%) outnumber rural residents (39%) for Thailand. The gender breakdown is similar for Malaysia, Indonesia, and Philippines, although males are under-represented for Thailand (43%) and Vietnam (44%). 

The highest proportion is found among individuals in the 30-35 years age bracket for all countries and the lowest from the retiree age group (≥60 years). The majority in Malaysia (64%), Vietnam (65%), and Philippines (43%) are high school educated, while those in Thailand (54%) and Indonesia (53%) are either uneducated or educated up to the primary level. The majority of respondents in Thailand (64%), Indonesia (61%), Vietnam (63%), and Philippines (58%) are non-government employees or self-employed, while the majority in Malaysia (65%) do not have any employment status (unemployed, student, homemaker, retired). About two-thirds in Malaysia (64%) and Thailand (62%) are married or living with a partner.

*Parameter estimates*

The ordered probit model is estimated for each sample by programming in R-4.4.0, using the numerical optimization package maxLik (Henningsen and Toomet, 2011). To determine whether to investigate all countries together as a sample or separate, our hypothesis is that socio-economic characteristics affect smoking behavior differently across countries. Denote the maximum log-likelihoods for the three countries and pooled sample as (logL_1_,logL_2_,logL_3_,logL_p_), with corresponding numbers of parameters (*k*_1_*,k*_2_*,k*_3_*,k*_p_), then the likelihood-ratio statistic LR=2(logL_1_,logL_2_,logL_3_,logL_P_) is chi-square distributed with (*k*_1_*,k*_2_*,k*_3_*,k*_p_) degrees of freedom. Using maximum log-likelihoods for Indonesia, Vietnam, and the Philippines and the pooled sample (with two country dummy variables), the null hypothesis that all slope coefficients are equal among the three countries is rejected (LR = 645.49, df = 22, p-value < 0.001). Following a similar procedure, equality slope coefficients is also rejected between Malaysia and Thailand (LR = 29.91, df = 12, p-value = 0.003). In sum, our statistical tests suggest differential effects of socio-economic characteristics on smoking behavior, which justifies separate analysis by country. 

Maximum-likelihood estimates are not presented and we only summarize the key results. Estimate for the threshold parameter (τ) is positive and significant at the 1% level of significance, suggesting success of the parameter in delineating the smoking categories for all countries. A negative or insignificant threshold parameter would have suggested misspecification of the model or improper categorization of smoking outcomes. Further, Wald (chi-square) tests suggest all slope coefficients are jointly significant, with a p-value < 0.001 for all countries. Indeed, the regression estimates suggest statistical significance of all but one variable in all countries.

With the current three-category model, the estimated coefficients exposure variables relates indirectly in signs to the probability of non-smoking, directly to the probability of daily smoking, but indeterminately to the probability of occasional smoking. Due to the parametric restrictions imposed for identification, the estimated coefficients reveal little about the magnitudes of effects of variables (Greene and Hensher 2010). To explore the effects of the roles of exposure variables, we calculate average marginal effects.

*Effects of exposure variables*

Tables 2-3 present marginal effects of exposure variables on smoking category probabilities for the separate countries. Household size is associated with smoking status in Malaysia and Vietnam only. Each additional member in the household reduces occasional and daily smoking likelihoods by 0.02 and 0.44 percentage point (henceforth, point(s)), respectively, whilst increasing non-smoking likelihood by about 0.46 point.

Place of residence is associated with smoking status in Thailand, Indonesia, and Philippines, although the associations are dissimilar. Urbanites in Philippines are more likely to smoke occasionally (0.15 point) and daily (1.42 points) than rural dwellers. Urbanites in Indonesia (1.83 points) and Thailand (2.45 points) are less likely to smoke daily but are more likely to be non-smokers (1.82-2.58 points) than others. Moreover, urbanites in Indonesia are 0.02 point more likely while those in Thailand are 0.13 point less likely to smoke occasionally than rural residents.

Gender differences are present as males are more likely to smoke either occasionally or daily than females across all countries. Even though Indonesian males are 62.04 points less likely to be non-smokers, they display higher propensities of occasional (7.85 points) or daily (54.19 points) smoking than their female cohorts. Similarly, whilst Vietnamese males are 46.30 points less likely to be a non-smoker, they are more likely to smoke occasionally (5.75 points) and daily (40.55 points) than their female counterparts.

Age is a factor, albeit with mixed relationships with smoking across countries. Younger middle-age (age 30-45 years) individuals in Malaysia and Thailand are more likely to smoke either occasionally or daily and are less likely to be non-smokers than their younger cohorts (age 15-29 years). Retirees (age ≥60 years) in Malaysia and Thailand are less likely to be occasional or daily smokers but are more likely to be non-smokers than others. However, a different scenario looms for countries like Indonesia, Vietnam, and Philippines as individuals 30 years and above consistently display higher propensities to smoke occasionally or daily and lower likelihoods of being non-smokers than their younger counterparts. 

Education level is associated with smoking status as higher educated individuals are less likely to smoke occasionally or daily but are more likely to be non-smokers. Tertiary (11.81 points) and high school (3.54 points) educated Malaysians are less likely to smoke daily than those with only primary school education; tertiary (0.62 point) and high school (0.11 point) educated Malaysians are also less likely to smoke occasionally. Instead, those with tertiary (12.43 points) or high school (3.65 points) education display higher propensities to be non-smokers than others.

Working sector is associated with smoking status as non-government or self-employed workers in all countries are consistently more likely to smoke either on occasional or daily basis and less likely to be non-smokers than those without employment status. Nonetheless, while government employees in Thailand, Vietnam, and Philippines display similar higher smoking tendencies than their unemployed counterparts, this association is not evident in Malaysia and Indonesia.

Marital status plays a role in Thailand as married individuals are 2.06 points more likely to be non-smokers and less likely to be occasional (0.11 point) and daily (1.96 points) smokers. This association is not present in Malaysia. 

**Table 1:** Variable Definitions and Sample Means (Standard Deviations)

Variable	Definition	Malaysia	Thailand	Indonesia	Vietnam	Philippines
Non-smokers	Not at all smoking now (includes former daily and never daily smokers)	0.77 (0.42)	0.79 (0.41)	0.66 (0.48)	0.78 (0.41)	0.76 (0.43)
Occasional smoker	Currently smokes tobacco on a less than daily basis (includes former daily and never daily smokers)	0.02 (0.14)	0.02 (0.14)	0.05 (0.22)	0.03 (0.17)	0.04 (0.20)
Daily smoker	Currently smokes tobacco on a daily basis	0.21 (0.41)	0.19 (0.39)	0.29 (0.45)	0.19 (0.39)	0.20 (0.40)
Continuous variable						
Household size	Number of persons in household	3.96 (2.20)	3.07 (1.61)	3.76 (1.70)	3.56 (1.88)	4.47 (2.29)
Binary variables (Yes = 1; No = 0)						
Urban	Resides in urban area	0.49	0.61	0.49	0.49	0.40
Male	Gender is male	0.49	0.43	0.48	0.44	0.50
Age 15−29	Age is 15-29 years (reference)	0.29	0.17	0.28	0.20	0.30
Age 30-45	Age is 30−45 years	0.33	0.30	0.38	0.33	0.34
Age 46-59	Age is 46−59 years	0.23	0.28	0.20	0.27	0.20
Age ≥60	Age is ≥60 years	0.16	0.24	0.14	0.20	0.16
Primary School	Possess primary school education or less (reference)	0.26	0.54	0.53	0.19	0.32
High School	Possess high school education	0.64	0.34	0.40	0.65	0.43
Tertiary	Possess tertiary education and beyond	0.10	0.12	0.07	0.17	0.25
Unemployed	No employment status (e.g. unemployed/student/homemaker /retired (reference))	0.65	0.30	0.35	0.28	0.37
Government	Government employee; others	0.09	0.07	0.03	0.09	0.05
Non-government	Non-government employee or self-employed	0.26	0.64	0.61	0.63	0.58
Married	Married or living with a partner	0.64	0.62	−	−	−
Sample size		4220	20586	8300	8985	11603

**Table 2:** Marginal Effects of Explanatory Variables on Smoking Category Probabilities: Malaysia, Thailand, Indonesia

	Probability (× 100) of		
Variable	Non-smoker	Occasional smoker	Daily smoker
	Malaysia		
Household size	0.46 (0.25)*	-0.02 (0.01)*	-0.44 (0.25)*
Urban	1.21 (1.11)	-0.04 (0.04)	-1.17 (1.07)
Male	-43.18 (1.15)***	3.51 (0.37)***	39.67 (1.11)***
Age 30-45	-2.94 (1.57)*	0.09 (0.05)*	2.85 (1.52)*
Age 46-59	-0.65 (1.74)	0.02 (0.06)	0.63 (1.69)
Age ≥60	4.58 (1.96)**	-0.17 (0.09)**	-4.40 (1.88)**
High School	3.65 (1.41)***	-0.11 (0.04)***	-3.54 (1.37)***
Tertiary	12.43 (1.78)***	-0.62 (0.14)***	-11.81 (1.66)***
Government	-1.74 (1.97)	0.05 (0.06)	1.69 (1.91)
Non-government	-4.56 (1.32)***	0.14 (0.05)***	4.42 (1.28)***
Married	1.25 (1.32)	-0.04 (0.04)	-1.21 (1.28)
	Thailand		
Household size	0.10 (0.15)	-0.01 (0.01)	-0.10 (0.15)
Urban	2.58 (0.50)***	-0.13 (0.03)***	-2.45 (0.48)***
Male	-40.09 (0.56)***	3.27 (0.16)***	36.81 (0.54)***
Age 30-45	-1.53 (0.79)*	0.08 (0.04)*	1.45 (0.75)*
Age 46-59	-0.22 (0.83)	0.01 (0.04)	0.21 (0.78)
Age ≥60	2.88 (0.85)***	-0.16 (0.05)***	-2.73 (0.80)***
High School	5.09 (0.55)***	-0.26 (0.03)***	-4.82 (0.52)***
Tertiary	13.83 (0.64)***	-0.97 (0.08)***	-12.85 (0.58)***
Government	-3.67 (1.23)***	0.18 (0.06)***	3.49 (1.18)***
Non-government	-7.04 (0.63)***	0.41 (0.05)***	6.63 (0.59)***
Married	2.06 (0.55)***	-0.11 (0.03)***	-1.96 (0.53)***
	Indonesia		
Household size	0.21 (0.21)	0.00 (0.00)	-0.21 (0.21)
Urban	1.82 (0.73)**	0.02 (0.01)	-1.83 (0.74)**
Male	-62.04 (0.90)***	7.85 (0.39)***	54.19 (0.88)***
Age 30-45	-4.11 (0.93)***	-0.03 (0.02)	4.13 (0.94)***
Age 46-59	-5.81 (1.07)***	-0.08 (0.04)**	5.88 (1.09)***
Age ≥60	-2.22 (1.21)*	-0.02 (0.02)	2.24 (1.23)*
High School	6.38 (0.81)***	0.08 (0.04)**	-6.45 (0.82)***
Tertiary	12.17 (1.51)***	-0.26 (0.11)**	-11.91 (1.42)***
Government	-2.03 (2.19)	-0.02 (0.03)	2.05 (2.21)
Non-government	-9.63 (1.05)***	0.19 (0.07)***	9.44 (0.99)***

Note: Asymptotic standard errors in parentheses. *** p < 0.01, ** p < 0.05, * p < 0.10

**Table 3:** Marginal Effects of Explanatory Variables on Smoking Category Probabilities: Vietnam, Philippines

	Probability (× 100) of		
Variable	Non-smoker	Occasional smoker	Daily smoker
	Vietnam		
Household size	0.43 (0.20)**	-0.02 (0.01)**	-0.41 (0.19)**
Urban	0.00 (0.70)	0.00 (0.04)	0.00 (0.66)
Male	-46.30 (0.79)***	5.75 (0.34)***	40.55 (0.76)***
Age 30-45	-7.97 (1.04)***	0.37 (0.06)***	7.60 (0.99)***
Age 46-59	-10.21 (1.09)***	0.43 (0.06)***	9.78 (1.04)***
Age ≥60	-7.32 (1.21)***	0.30 (0.05)***	7.02 (1.17)***
High School	7.11 (0.92)***	-0.30 (0.04)***	-6.81 (0.88)***
Tertiary	12.06 (1.08)***	-0.85 (0.12)***	-11.21 (0.98)***
Government	-3.63 (1.65)**	0.16 (0.07)**	3.47 (1.59)**
Non-government	-9.82 (0.94)***	0.61 (0.09)***	9.21 (0.87)***
	Philippines		
Household size	-0.19 (0.15)	0.02 (0.02)	0.17 (0.13)
Urban	-1.58 (0.73)**	0.15 (0.07)**	1.42 (0.66)**
Male	-32.00 (0.77)***	4.47 (0.22)***	27.54 (0.68)***
Age 30-45	-4.59 (0.94)***	0.44 (0.09)***	4.15 (0.85)***
Age 46-59	-6.20 (1.09)***	0.56 (0.10)***	5.64 (1.00)***
Age ≥60	-4.32 (1.22)***	0.39 (0.10)***	3.93 (1.11)***
High School	4.03 (0.80)***	-0.40 (0.08)***	-3.63 (0.72)***
Tertiary	11.00 (0.88)***	-1.24 (0.13)***	-9.76 (0.77)***
Government	-6.81 (2.02)***	0.58 (0.15)***	6.24 (1.88)***
Non-government	-9.43 (0.85)***	1.07 (0.12)***	8.36 (0.74)***

Note: Asymptotic standard errors in parentheses. *** p < 0.01, ** p < 0.05, * p < 0.10

## Discussion

Motivated by the empirical literature suggesting different driving forces of non-smoking, occasional smoking, and daily smoking, this study is first in kind to examine the socio-demographic factors associated with these multiple smoking outcomes based on data from standardized large-scale national surveys of five ASEAN countries. Several observations are noted. First, results that Malaysian and Vietnamese households with more family members face lower smoking likelihoods than otherwise support findings from previous studies that household size negatively affects demand for cigarettes (Tan et al., 2009; Buonanno and Ranzani, 2013). Individuals from households with more family members may face tighter budget constraints, thus leaving less for non-essential items such as cigarettes (Aksoy et al., 2019). Nonetheless, it is interesting to note the disassociation between household size and smoking status for the remaining countries.

Second, the association between area of residence and smoking status is mixed for different countries as urbanites in Philippines and rural residents in Thailand and Indonesia are more likely to smoke occasionally and daily than others. These findings suggest that while smoking behavior may stem from social norms, peer pressure, and more intense media advertising within the urban surroundings in Philippines, limited health literacy and cultural acceptability may also contribute to the smoking occurrence in rural Thailand and Indonesia (Kusumawardani et al., 2018). The health authorities in these countries should therefore target anti-smoking programs at these specific communities to discourage cigarette consumption.

Third, males are consistently more likely to smoke, occasionally or daily, than females across all countries. This finding corroborates previous studies globally as well as from the countries considered in the current study, albeit with dissimilar data sets (Tan et al., 2009; Punzalan et al., 2013; Ko and Pumpaibool 2016; Kusumawardani et al., 2018). As such, anti-smoking measures in affecting behavioral change may include ameliorating the male perception towards smoking across all countries. Such measures could include reversing the perception that smoking imparts sexuality to females as well as a machismo appearance to males. Efforts could also be made to highlight the possibilities of erectile dysfunction and impotency due to smoking instead (Verze et al., 2015).

Fourth, age is associated with smoking status across countries. This occurs as individuals in all older age groups (age 30−45 years, age 45−59 years, age ≥60 years) in Indonesia, Vietnam, and Philippines are consistently more likely to smoke either occasionally or daily than their younger (age 15−29 years) cohorts. This worrying trend suggests that unless proper mitigative actions are undertaken, the health and economic burdens of tobacco smoking in these countries will continue to rise as the population ages. Meanwhile, findings that Malaysians and Thais in the retiree age group (age ≥60 years) are less likely to smoke either occasionally or daily than others may allude to changing smoking patterns in these countries. Previous anti-smoking measures may have effectively lowered the smoking likelihoods of those in these age group. 

Fifth, it is worth noting that education dampens occasional and daily smoking likelihoods whilst increasing non-smoking propensities in all countries. Higher educated individuals may possess better cognitive skills and understanding of the relationship between health behavior and health (Tan et al., 2009). Therefore, public health authorities in the region should further enhance programs that advocate education and schooling as a means to reap the positive external effects on the long-term health of its citizens. Human capital investments in education may even offset the long run health costs arising from cigarette smoking.

Sixth, as non-government or self-employed workers in all countries are more likely to smoke occasionally or daily than unemployed persons, gainful employment may proxy higher disposable income to afford smoking indulgence (Azagba and Sharaf, 2013). Further increases in cigarette excise taxes can therefore raise tax revenue and dampen affordability among the working class. Meanwhile, the inverse association between working and smoking statuses also suggests that workplace anti-smoking restrictions may be an effective tool against smoking in ASEAN countries. 

Last, the outcome that marriage is associated with higher non-smoking likelihoods in Thailand corroborates previous studies that marriage may be a source of social support with protective health effects (Chassin et al., 2010). Nevertheless, while marital status information is unavailable for other countries and given the non-association between this variable and smoking status in Malaysia, it may still be viable to stress family relationships as an important reason to quit smoking as a “no-regret” anti-smoking policy.

Several limitations are acknowledged. Income was omitted although it is traditionally included in cigarette consumption studies. This information was not collected in the GATS. In its absence, we posit that education level (primary and below, high school, tertiary) may capture the role of income (wealth) as suggested by other studies on smoking behavior (Wu et al., 2019). Nonetheless, we note interpretation of the results with care. The cross-sectional data type of GATS also does not allow for inter-temporal occurrences to be taken into consideration, nor does it allow investigation of the dynamics of smoking behavior such as the role of addiction. Further studies should replicate our analysis based on longitudinal data to examine the robustness of our findings. Last, responses consist of self-reports of smoking behavior. Biochemical verification of tobacco usage may provide for more accurate validation of self-report smoking status. 

This study concludes that no exact “one size fits all” approach is available to address the cigarette smoking problem among ASEAN countries. The most effective public health strategy for smoking prevention remains a portfolio of systemic and targeted interventions designed to address the smoking issue within various country settings. Findings of this study can contribute to public health deliberations within the ASEAN population with insights on the characteristics of the different smoking populations. From these outcomes, it may be possible to direct health and educational programs for smoking prevention and cessation to target groups in the ASEAN region.

## Author Contribution Statement

Both authors contributed to the idea, design, analysis, and write-up of the study and approved the final version of the manuscript.
